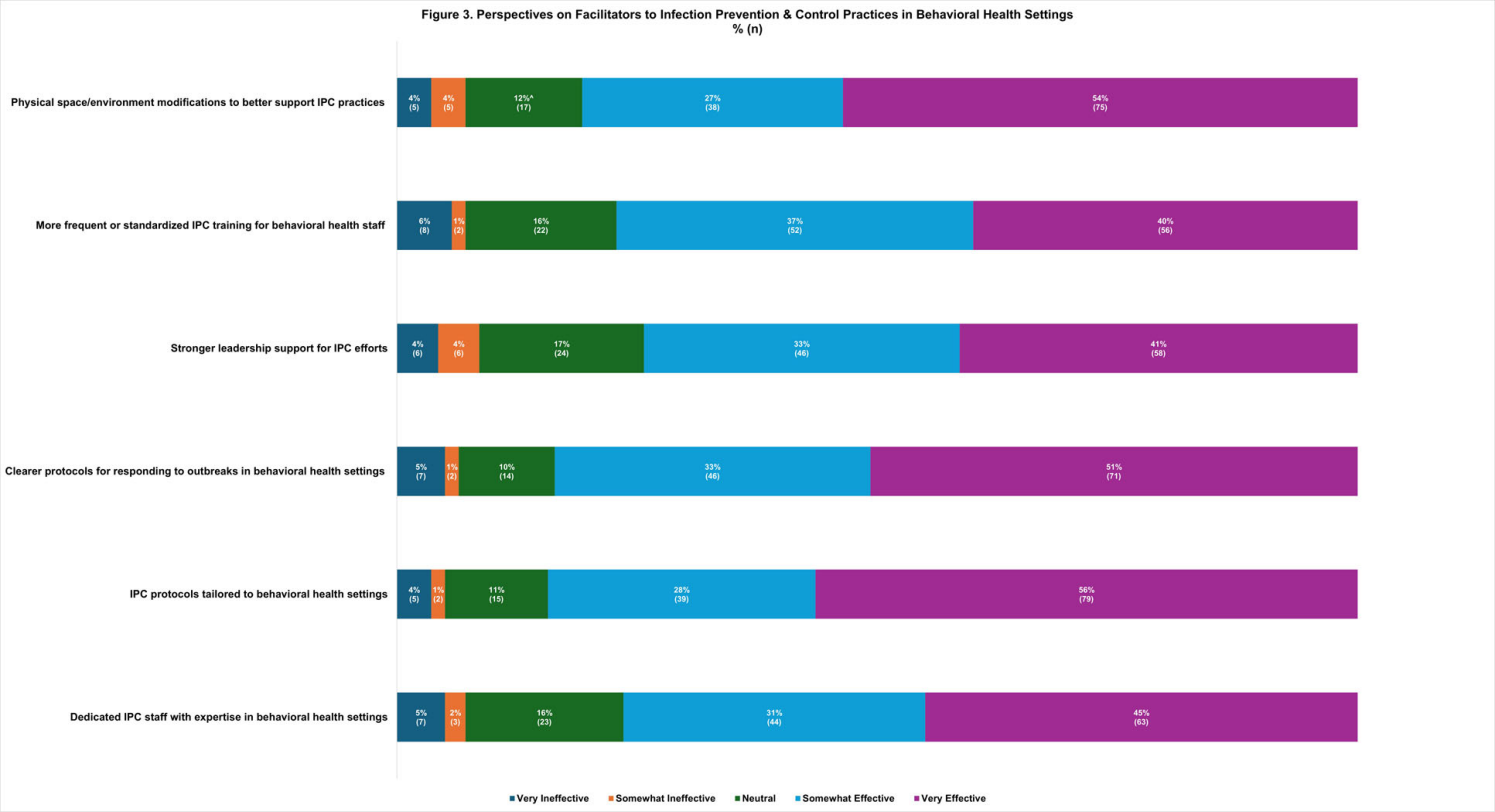# 30 A Proactive Risk Assessment of Central Line Care Following a CLABSI Cluster

**DOI:** 10.1017/ash.2026.10474

**Published:** 2026-06-23

**Authors:** Isabelle Boullier, Thomas Head, Kevin Gibas

**Affiliations:** 1 Brown University School of Public Health; 2 Rhode Island Hospital

## Abstract

**Background:** Infection prevention and control (IPC) is essential for patient safety across all healthcare settings, yet implementation varies by patient population and care environment. Behavioral health settings present unique and underrecognized IPC challenges related to the care environment and the unique nature of psychiatric care. Patient behaviors and cognitive conditions may hinder adherence, while safety- and therapy-focused features complicate implementation of IPC best practices. Evidence tailored to behavioral healthcare environments remains limited, underscoring the need for contextually appropriate, patient-centered strategies. **Method:** We conducted a cross-sectional, web-based survey to examine barriers and facilitators influencing implementation of IPC practices in behavioral health settings. The survey was distributed to U.S. and Canadian healthcare organizations participating in the SHEA and APIC Research Networks. Eligible respondents included individuals responsible for IPC programs or institutional policy development. The survey was developed using REDCap and distributed via email through the APIC/SHEA Research Networks over six weeks. Participation was voluntary, anonymous, and uncompensated. **Result:** A total of 140 respondents completed the survey (response rate: 13%, n=140/1095). Respondents were predominantly hospital epidemiologists (17%, n=21) and infection preventionists (76%, n=96) representing public (34, n=42%), academic/teaching (33, n=41%), non-profit private (25%, n=31), and for-profit private healthcare systems/hospitals (7%, n=9). 81% of respondents reported implementing IPC mitigation plans for outbreaks in the past year, with COVID-19 (76%, n=107), Influenza (52%, n=73), and Norovirus (37%, n=52) being the most cited pathogens. The most identified barriers to IPC implementation/adherence were patient factors (89%, n=124) and environmental constraints (79%, n=111). More than 75% of respondents reported that patient adherence to transmission-based precautions, isolation practices, masking, and hand hygiene were “moderately” or “very” challenging. Participants identified the most effective facilitators to effective IPC in behavioral health settings as IPC protocols tailored to behavioral health, clear outbreak-response guidance specific to behavioral health, and physical space or environmental modifications to better support IPC practices. **Conclusion:** This study highlights the substantial and distinct barriers to implementing infection prevention and control best practices in behavioral health settings, driven by the unique care environment and patient population. Despite having foundational IPC infrastructure in place, organizations face persistent challenges with practical implementation, including patient adherence to precautions and isolation, as well as environmental and physical design constraints. Our findings underscore the need for context-specific IPC protocols, targeted environmental adaptations, and dedicated IPC expertise to improve patient safety in these often underrecognized healthcare settings.

**Figure:**
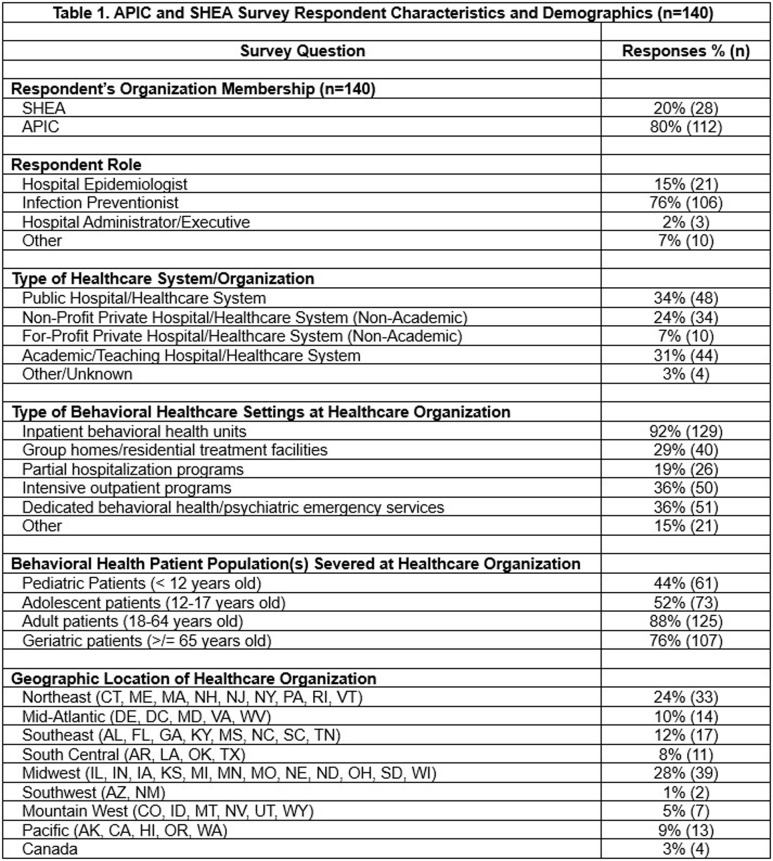

**Figure:**
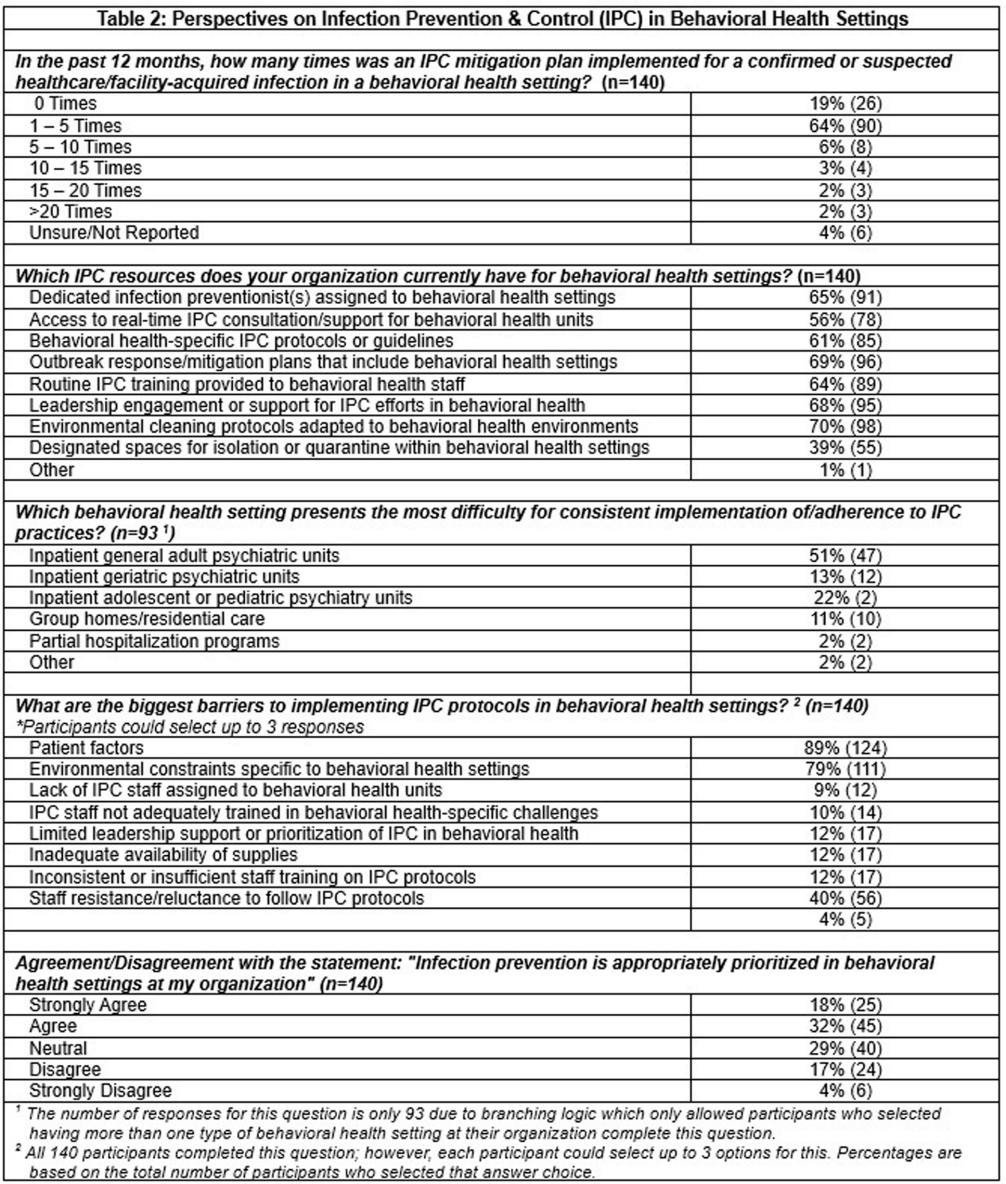

**Figure:**
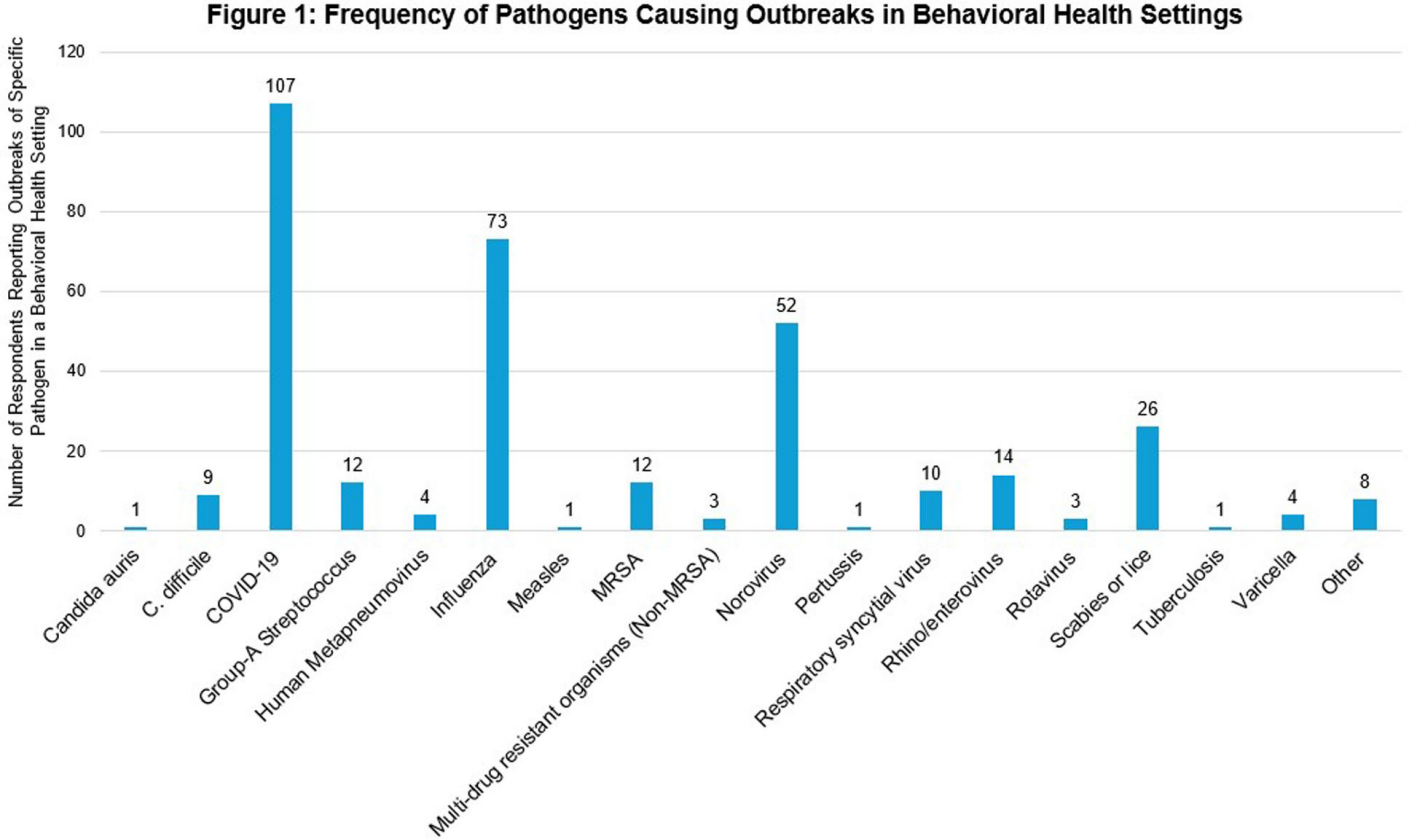

**Figure:**
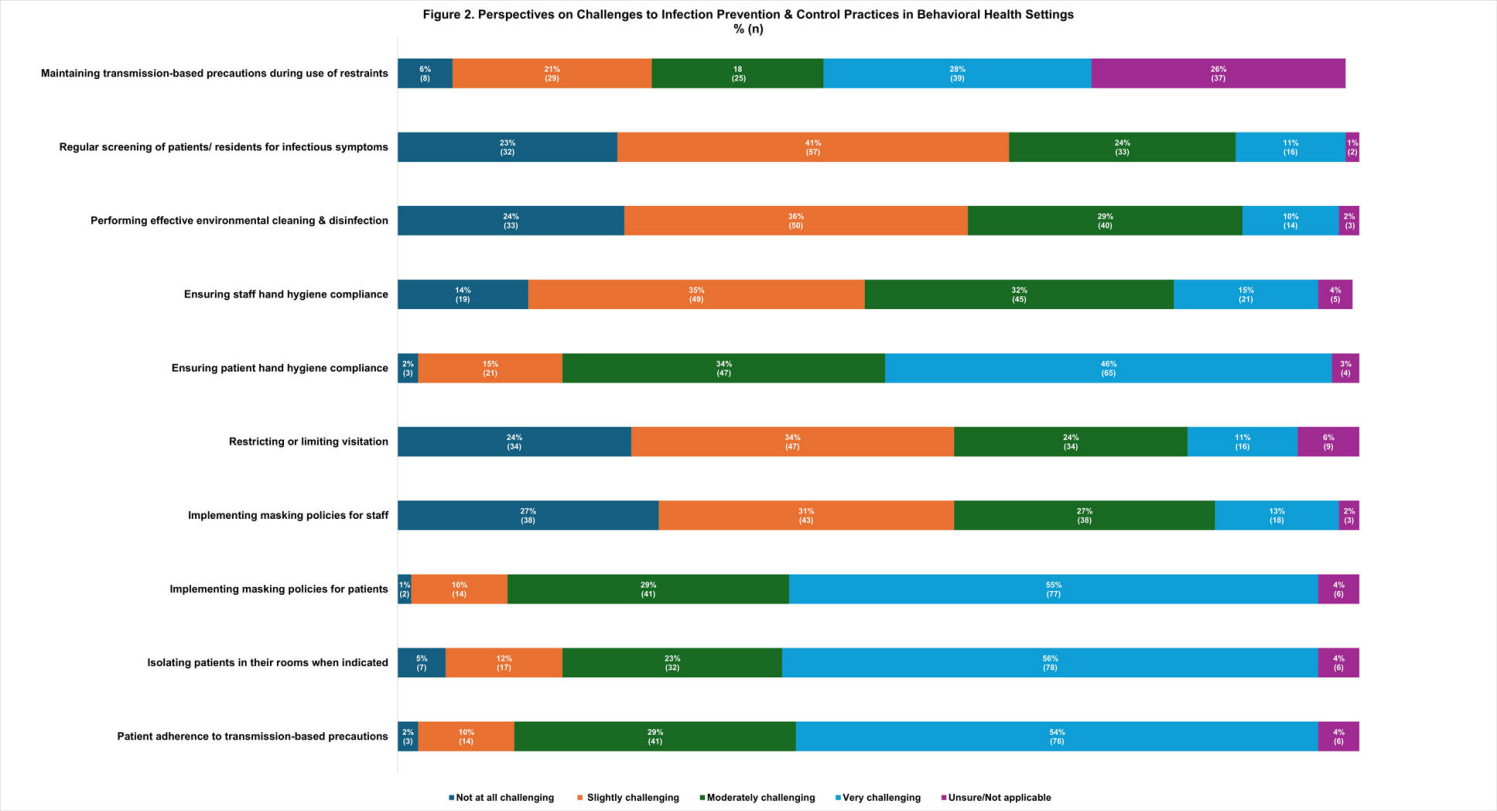

**Figure:**